# Treatment of Emergencies

**Published:** 1887-08-06

**Authors:** 


### Till the Doctor Comes.

Inquiries and Communications for this column should be addressed "Physician," care of the Editor of The Hospital.]

### XXIII.—TREATMENT OF EMERGENCIES.

(*Continued.*)

*Foreign Substances in Windpipe.*—These are sometimes drawn into this tube by a sudden breath whilst eating, as from laughing, etc., or when a substance is being carried in the mouth. It is no easy matter to dislodge them, and they will give rise to difficult breathing, distressing paroxysms of cough, etc. The patient may be placed on his stomach, with the head downwards and the back well slapped. This is seldom successful, and surgical assistance is almost always necessary.

A large piece of meat or food may lodge in the swallow, so as to shut off the entrance to the windpipe; it can usually be here reached by the finger and removed.

*Stings by venomous Insects, as Gnats, Wasps, etc.*—Remove the sting if present. Vinegar and water, dilute ammonia, or eau de cologne and water, will give relief: moist soil immediately applied is also said to remove the pain at once. Where there are many wasp-stings, some stimulant may be necessary. After a snake or adder bite, a band should be tied tightly round the limb above the bite, to prevent entrance of the poison into the system, and the place should be well burnt by a red-hot iron. A free use of stimulants is essential.

*Stings from Plants, Nettles, etc.*—Apply dilute solution of ammonia or of carbonate of soda. This will give great relief if done at once.

*Bite by Dog.*—Wash the wound well and encourage bleeding. It is best then to freely apply nitric acid to it, as this is a liquid, and gets more freely to every corner of the wound; but if this would necessitate delay, use a hot iron, or lunar caustic. If the dog is known to be rabid, the hot iron should be used; or if the wound is on the finger, it is better at once to amputate it. An absurd superstition is held in many country places, that if the dog is killed the person bitten will not have hydrophobia; this, of course, is a most ignorant and silly notion. It is much more satisfactory to the mind of the person bitten to keep the dog under observation, to see if he develops rabies.

*Sunstroke.*—Remove into a cold place. Apply cold douche to the head and cold sponging to the body. Fan the body. Give small quantities of cool water, but in this be very careful; and also in applying the cold douche, do not go to extremes.

*Burns.*—If slight and simply occasioning redness, dust well with flour, and wrap plenty of cotton-wool round the part. If it has gone a stage farther, and blisters have formed, spread some vaseline, zinc ointment, or cold cream on strips of lint; prick the blisters in several places with a needle to let out the fluid, wrap the ointment round it, apply plenty of cotton-wool, and a bandage. Any very severe burn may be dressed in the same way till help arrives. The great point is to exclude all access of air. Give stimulants if necessary, and keep the patient warm. It cannot be too widely known, that the best way of putting out the flames, when a person's clothes have caught fire, is by taking the hearth-rug, a great-coat, or any similar article that is handy, and wrapping it closely round him, or rolling him in it, so as to entirely prevent the access of air.

*Burns by Acids.*—Bathe the parts with an alkaline fluid like dilute ammonia, or carbonate of soda in solution (this is generally at hand), and afterwards dress as a *burn.*

*Burns by Lime.*—Bathe the part with vinegar and water. If lime gets into the eye, it will do an immense amount of mischief, and always requires medical advice, or permanent mischief will result. Till advice can be procured, weak vinegar and water must be freely applied by running it over the eye.

*Lightning Stroke*—Apply cold to the head, and, if necessary, warmth to the extremities; rub the limbs well, and give some stimulants, as soon as the patient can swallow.

*Cold and Frostbite.*—If a person is suffering very severely from the effects of cold, the temperature must only be raised very gradually. He must be brought into a cold room, his clothes removed, and blankets wrapped round him: the limbs and body must be gently and continuously rubbed, first with ice, then with dry flannel, or the hand. When he can swallow, warm drinks may be given, and later on stimulants. If a part only of the body gets frostbitten, as the nose, it is known by its first becoming blue and congested, and afterwards yellowish and tallowy; sensation is lost in it, and the sufferer may not know what mischief is caused. Rub the part first with snow, and so on as above.

*Foreign Bodies in Nose and Ear.*—Leave them alone. It is usually difficult for a doctor to remove them, and if an unskilled person attempts it, he will render the doctor's task much more difficult, probably impossible. Gentle syringing may alone be tried on the ear.

*Swallowing foreign Bodies, as Coins, Needles, etc.*—Do not be in a hurry to give castor-oil, etc.; in fact, do just the reverse. Keep the bowels confined, and give glenty of suet-pudding, gruel, and such like food. By this means the needle or other object is more likely to get imbedded in the mass, and to do no injury to the bowels. If the substance sticks before reaching the stomach, try the effect of swallowing large draughts of water, etc. If it is too large to pass into the stomach, and cannot be reached with the finger, surgical aid must be sought.

(*To be continued.*)

